# Physicochemical, functional, and nutritional characteristics of stabilized rice bran form tarom cultivar

**DOI:** 10.1002/fsn3.407

**Published:** 2016-07-11

**Authors:** Ali Rafe, Alireza Sadeghian, Seyedeh Zohreh Hoseini‐Yazdi

**Affiliations:** ^1^ Department of Food ProcessingResearch Institute of Food Science and Technology (RIFST)PO Box 91735‐147, MashhadIran; ^2^ Quality Responsible of Aftab Nokhiz Hezar Masjed CompanyMashhadIran

**Keywords:** functional, nutritional, physciochemical, rice bran, stabilization

## Abstract

Extrusion is a multistep thermal process which has been utilized in a wide spectrum of food preparations. The effect of extrusion processing on the physicochemical, nutritional, and functional properties of Tarom cultivar rice bran was studied. However, the color of rice bran was improved by extrusion processing, but the protein content was reduced in the stabilized rice bran, which can be related to the denaturation of protein. Extrusion had also a reduction significant effect on the phytic acid as well as vitamin E in rice bran. However, the content of niacin, riboflavin, pantothenic acid, and folic acid remained unchanged, but the dietary fiber was enhanced which has beneficial health effect on human consumption. In comparison with unstabilized rice bran, water holding capacity was enhanced, but the oil absorption capacity was reduced. Foaming capacity and foaming stability of extruded rice bran was more than that of untreated rice bran, although they were less than that of rice bran protein concentrate/isolate. In general, the extrusion process improves some functional and nutritional properties of rice bran which are valuable to industrial applications and have potential as ingredient in food to improve consumer health.

## 1 Introduction

Rice bran is an inexpensive by‐product of raw rice (*Oryza sativa L*.), which is widely cultivated in many countries all over the world (Sumantha et al., 2006). The global production of paddy rice in 2014 was over 738 million metric tons (MMT), which provides approximately 70 MMT of bran (FAO, 2014). However, rice bran possesses important components such as proteins and phytochemicals that supply beneficial health effects on the human body; it is mainly used for animal feed. Being high in protein, particularly the essential amino acid lysine, soluble and insoluble dietary fiber, it exhibits high nutritional value for human consumption (Sánchez, Quintero, & González, 2004). Thus, these properties are considered a healthy functional food, which has hypoallergenic, hypocholesterolemic and antioxidative properties (Kawase, Matsumura, Murakami, & Mori, 1998; Shih & Daigle, 2000). Indeed, the healthy effects of rice bran have encouraged many researchers to study its ability to be used as an important source of nutrients in food ingredients (Faria et al., 2012; Imsanguan et al., 2008; Lilitchan et al., 2008; Parrado et al., 2006; Renuka & Arumughan, 2007; Xu & Godber, 2000).

In spite of the benefits of rice bran, due to the oxidation of edible oil, it cannot be directly utilized for human consumption and needs further processing. Since, rice bran is rich in lipids and possesses lipoxygenase which make it susceptible to hydrolytic rancidity, it needs enzymatic inactivation instantly prior to any application to avoid fatty acid liberation and to extend its shelf life and permit its commercialization for human consumption (Wada, 2001). The inactivation of enzymes can be achieved by heating to high temperatures for a short time. Extrusion cooking is one of the most suitable procedures to stabilize rice bran (Ramezanzadeh et al., 1999; Randall et al., 1985; Tribelhorn, Cummings, & Kellerby, 1979). It is a multistep, multifunctional thermal process which utilizes high heat, pressure, and shear (Altan, McCarthy, & Maskan, 2008; Kim, Tanhehco, & Ng, 2006; Singh, Gamlath, & Wakeling, 2007). The extrusion process has many advantages such as low cost, high speed, efficiency, flexibility, unique product shapes, and energy savings over other common processing methods (Faraj, Vasanthan, & Hoover, 2004). Some chemical changes including gelatinization of starch molecules, cross‐linking of proteins, and flavor production have occurred during extrusion. Enochian, Saunders, Schultz, Beagle, and Crowley (1981) have conducted the economically feasibility study for stabilization of rice bran by extrusion cooking and have shown that it would be practical in certain developing countries. Since, in a few countries such as Iran, stabilized rice bran (SRB) is not broadly available at supermarkets, its stabilization can be suitable for further applications such as food supplement (Silva, Sanches, & Amante, 2006).

However, many works have been accomplished on the stabilization of rice bran and the effect of extrusion on some functional properties of food have been studied (Altan et al., 2008; Singh et al., 2007), but they still lack of knowledge concerning the stabilized rice bran and surveying of its functional and nutritional properties. Therefore, the main aim of the current work is to compare the functional, nutritional and biophysical characteristics of rice bran before and after stabilization. Certainly, these evaluations can be vital to utilize rice bran as a food ingredient for the human diet. In the view point of competitive market aspect, when more added value was found for rice bran, and more scientific knowledge concerning with its health benefits was achieved, it is expected that more industrial interest in processing of rice bran will be more valuable for human consumption than its use as animal feed.

## 2 Materials and Methods

### 2.1 Materials

The commercially dried rough rice of Tarom cultivar was kindly provided from Ricelands of Dargaz (Dargaz, Razavi Khorasan Province, Iran). It was packed in a hermetic plastic bag and kept in a refrigerator (3°C) until further processing. The ingredients were all of analytical grade and purchased from Merck or Sigma‐Aldrich Co. (St. Louis, MO).

### 2.2 Stabilization of rice bran

Rice bran was stabilized by a twin‐screw co‐rotating, self‐cleaning extruder (DS56‐X, Jianan Saixin Machinery Co., Jinan, China), with length/diameter ratio of 25, screw speed up to 600 rpm. Based on our preliminary experiments; temperature, screw speed, and throughput were selected as 130°C, 300 rpm, and 450 g/min, respectively. The moisture content of the rice bran was adjusted to 13%. Then, bran samples were immediately packed in polyethylene bags and stored at 4°C until analyses were completed.

### 2.3 Physicochemical characteristics

The protein, fat, moisture, and ash of unstabilized rice bran (USRB) were measured by the methods of Association of Official Analytical Chemists Society (AOAC International, 2000). Soluble dietary fiber was also determined with some modifications by the procedure of Zhang, Bai, and Zhang (2011). The carbohydrate content was calculated by subtracting the amount of other ingredients from 100.

The physical properties of samples were determined by bulk density and solubility index. Bulk densities of rice bran before and after 100‐tapping were determined based on the method of Okaka and Potter (1977). An aliquot of 50 g of rice bran powder was poured into a 100‐ml graduated measuring cylinder. The cylinder was tapped several times (100) on a lab bench to approach a constant volume. Then, the bulk density (ρ_b_) values were calculated prior to and after tapping and were given as g/ml. The solubility of bran was also determined by preparing 10% solution of rice bran in distilled water. The solutions were centrifuged at 5,000*g* for 5 min and the supernatant was removed. The insolubility index was determined in percent.

### 2.4 Appearance characteristics (image processing)

Image acquisition and processing of processed and untreated rice bran was performed based on the published literature (Abdollahi Moghaddam, Rafe, & Taghizadeh, 2015). Briefly, the images were captured by a color digital camera (Canon EOS 1000D, Taiwan) with resolution (2272 × 1704 pixels) in a wooden black box, and they were saved on a computer with software (Canon Utilities Zoom Browser EX version 6.1.1) in JPEG format. The image processing was accomplished by the Image J software (National Institutes Health, Bethesda, MD) after improving the image quality. Then, RGB images were converted into L*a*b* units in which L* is lightness (0 (black) to 100 (white)), a* is varied from red (+60) to green (−60) index, and b* is ranging from yellow (+60) to blue (−60). The total color change (ΔE) was also determined by the following equation:

(1) $$\Delta E=\sqrt{{\left(L_{2}^{*}-L_{1}^{*}\right)}^{2}+{\left(a_{2}^{*}-a_{1}^{*}\right)}^{2}+{\left(b_{2}^{*}-b_{1}^{*}\right)}^{2}}$$

where, subscribes 1 and 2 are before and after processing with extrusion cooking, respectively.

### 2.5 Functional properties

#### 2.5.1 Protein solubility

The protein solubility was determined by Folin reaction through the method of Lowry (Lowry, Rosenbrough, Farr, & Randall, 1951). In order to measure the protein solubility of rice bran at different pH levels, the appropriate amount of powder was dispersed in distilled water and pH was adjusted in range of 2.0–10.0 by NaoH or HCl 0.1 N. Then, the solution was dispersed gently at ambient temperature for 15 min. The dispersed samples were centrifuged at 1,200*g* for 20 min and the supernatant was decanted and the protein was measured according to Lowry method at 750 nm (UV‐visible spectrophotometer, Shimadzu, UV‐160A, Japan) (Frolund, Palmgren, Keiding, & Nielsen, 1996; Gerhardt, Murray, Wood, & Krieg, 1994; Lowry et al., 1951).

#### 2.5.2 Foaming capacity and stability

The foaming capacity and stability were evaluated by Kato procedure with some modifications (Kato, Takahashi, Matsudomi, & Kobayashi, 1983). The unstabilized and stabilized rice bran samples were prepared in distilled water (1% w/v) and the pH was adjusted to 5.0–8.0. Foaming capacity was compared with the volume of foams instantly after 1 min of mixing with a homogenizer at 10,000*g*. Then, it was stated by the following equation:

(2) $$Foamingcapacity=\frac{\text{Total volume}-\text{Drianage volume}}{\text{Initial volume of 100 ml}}$$

Foaming stability was also determined as the foam volume after 10 min and calculated as:

(3) $$Foamingstability=\frac{V_{0}\times 100}{\Delta V}$$

where *V*_0_ is the initial volume at the first time and *ΔV* is the change in volume of foam during the time interval *t* (10 min).

#### 2.5.3 Water and oil absorption capacity

The water absorption capacity of samples was evaluated by the Sosulski method (Sosulski et al., 1976). The unstabilized and stabilized samples of rice bran was dispersed in distilled water (12% w/v) and kept for 1 hr at room temperature (~25°C). Then, samples were centrifuged at 3,000*g* for 25 min. After 30 min, the supernatant was removed and the residues was dried at 110°C for at least 1 day and weighed. The water absorption was expressed as ml of water retained by insoluble fraction per gram of total rice bran (Petruccelli & Anon, 1995). For the oil absorption, it was determined by the method of Lin, Humbert, and Sosulski (1974) with some modifications. Aliquots of rice bran (0.5 g) was dispersed in a commercial corn oil and remained in a magnet stirrer for 30 min with gentle agitation. Then, the free oil was decanted and absorbed oil was measured by difference. The oil absorption capacity was stated as ml of absorbed oil per gram of sample.

### 2.6 Nutritional properties

Phytic acid content of rice bran before and after stabilization was measured according to the procedure of Garcia‐Estepa, Guerra‐Hernandez, and Garcia‐Villanova (1999) with slight modifications. In brief, 0.5 g of milled samples were extracted under magnetic agitation with 40.0 ml of sodium sulfate solution (10% in 0.4 mol/L HCl) for 3 hr at ambient temperature. The dispersion was centrifuged at 5000 g for 30 min. The supernatant was mixed with 10 ml of HCl (0.4 mol/L), 10 ml of FeCl_3_ (0.02 mol/L), and 10 ml sulphosalicylic acid and the tubes were shaken. Then, the tubes were placed in a boiling water bath for 15 min. The cooled samples were centrifuged at 5000 rpm for 10 min. The residue was removed, but the supernatant were diluted and the pH was adjusted to 2.5 by adding glycine. The solution was heated to 80°C and titrated with EDTA (50 mmol/L). The phytic acid content was calculated based on the atomic ratio of Fe/P 4:6. The colorimetric method was also used to determine the phytic acid content, particularly for the stabilized samples which are out of the accuracy of the titration method. The colorimetry was carried out by the AOAC method at 420 nm.

Vitamin E was determined as α‐tocopherol acetate by the method Shin, Godber, Martin, and Wells (1997). Breifly, the rice bran was saponified in a water‐methanol medium and extracted by petroleum ether. Vitamin E was determined as DL‐α‐tocopherol by HPLC using a UV‐detector and calculated by means of external standard. The specification of HPLC system was included as column (Macher/Nagel Nucleosil CN, 5 μm in dimension 300*8*4 mm), elution solvent (n‐hexan with 1% isopropanol), flow rate (2 ml/min), integrator (Sigam 15 Chromatography Data Station, Perkin‐Elmer), and wavelength (286 nm).

The extraction of B vitamins was performed similar to that of Arella, Lahely, Bourguignon, and Hasselmann (1996) with slight modifications. Vitamins B_1_ (thiamine) and B_2_ (riboflavin) were also determined by Arella et al. (1996) methods. Vitamin B_3_ (niacin) was analyzed according to spectrometric AOAC method (AOAC International, 2000) and vitamin B_5_ (pantothenic acid), B_6_ (pyridoxine) and B_9_ (folic acid) were measured by the Tuncel, Yılmaz, Kocabıyık, and Uygur (2014) and AOAC method, respectively.

### 2.7 Statistical analysis

The physicochemical, functional, and nutritional characteristics of rice bran before and after stabilization were evaluated in triplicates and data were averaged. Data were presented in mean ±  SD. The Duncan's multiple range test at 5% level was applied to evaluate significant differences between the means of each treatment.

## 3 Results and Discussion

### 3.1 Physicochemical properties

The chemical composition of processed and untreated rice bran by extruder is presented in Table 1. It can be seen that rice bran is a rich source of protein (15%), fat (22%), and dietary fiber (27%). Indigestible ingredients such as cellulose, hemicellulose, oligosaccharides, and pectin as well as lignin and waxes are altogether considered as dietary fibers. The dietary fibers play an important role in a healthy food and diet. Our findings showed that processed and untreated rice bran had valuable fiber of approximately 27%, which was more than that of other cultivars of rice mentioned in the literatures (Kim, Byun, Cheigh, & Kwon, 1987; Vasanthan, Gaosong, Yeung, & Li, 2002; Zhang et al., 2011). Although the protein and fiber content of Tarom cultivar rice bran was similar to the previous work (Abdul‐Hamid & Luan, 2000), the minerals and lipids of Tarom rice bran was more than that of their results, which may be more susceptible to hydrolytic rancidity. The results showed that the protein and mineral contents of Tarom cultivar rice bran was in agreement with protein (15.32%) and mineral (11.32%) of the unstabilized defatted rice bran (Gnanasambandam & Hettiarachchy, 1995). In comparison of USRB with stabilized rice bran (SRB), it can be found that all the components except minerals and lipids varied during extrusion. In fact, the extrusion process by applying high heat and shear reduced the moisture, protein, and fiber, but lipids and minerals remained statistically unchanged (Table 1). However, the carbohydrate was improved for SRB samples, which can be related to the calculation method. The reduction of protein content in the SRB may also be attributed to the denaturation protein at the high shear and temperature during extrusion.

**Table 1:** Proximate analysis of treated and untreated rice bran by extruder

Components	USRB (%)^a^	SRB (%)^a^
Moisture	9.28 ± 0.25^a^	5.33 ± 0.57^b^
Lipids	22.40 ± 0.32^a^	22.07 ± 0.24^a^
Protein	15.00 ± 0.41^a^	8.50 ± 0.53^b^
Ash	10.07 ± 0.12^a^	10.05 ± 1.41^a^
Total dietary fiber	27.00 ± 0.02^a^	25.50 ± 0.01^a^
Digestible carbohydrates^b^	16.25^a^	28.55^b^
Biophysical properties		
Bulk density (before tapping), g/cc	0.32 ± 0.10^a^	0.44 ± 0.01^b^
Bulk density (after 100 tappings), g/cc	0.41 ± 0.20^a^	0.54 ± 0.01^b^
Solubility index, %	97.50 ± 0.10^a^	98.20 ± 0.10^b^

a All the experiments were carried out in triplicates. Statistical significant difference was presented in alphabetic order.

b Values were obtained by gravimetric method. SRB, stabilized rice bran; USRB, unstabilized rice bran.

Physical attributes including bulk density (ρ_b_) (before and after tapping) and solubility index of USRB and SRB are provided in Table 1. The bulk density is a key factor in food product packing. It can be seen that Tarom cultivar rice bran has ρ_b_ values less than that of SRB. Furthermore, by applying extrusion processing, rice bran solubility was improved. Esmaeili, Rafe, Shahidi, and Ghorbani Hasan‐Saraei (2016) have shown that the ρ_b_ of rice bran proteins from Tarom and Shiroodi cultivars were varied from 0.55 to 0.53 g/ml, respectively, which was similar to black bean flour and more than that of red kidney flour (Siddiq, Ravi, Butt, Harte, & Dolan, 2010). Moreover, the ρ_b_ values of SRB were less than casein (0.89 g/ml), which make it proper for formulation of the weaning foods (Chandi & Sogi, 2007; Onimawo & Egbekun, 1998).

The solubility index of USRB and SRB were significantly less than that of rice bran protein concentrate/isolate, which may be attributed to the high amount of fiber and insoluble matters in the studied rice cultivars (Esmaeili et al., 2016; Gnanasambandam & Hettiarachchy, 1995).

### 3.2 Appearance attributes

Color properties of rice bran before and after treatment with extrusion are surveyed. The results showed that lightness index (L*) was reduced by extrusion processing from 57.02 ± 2.3 to 43.17 ± 2.2. However, our data were less than that of L value of Lemont and Nato cultivars from Louisiana, which may be related to the different cultivar and the Hunterlab method they used in color measurement (Tao, Rao, & Liuzzo, 1993). Nevertheless, the microwave heating has induced reduction in lightness of stabilized rice bran and stabilization process can be maintained color of SRB during preservation in 6 months (Bagchi, Adak, & Chattopadhyay, 2014; Tao et al., 1993). Indeed, extrusion cooking can modify appearance of rice bran to have great customer acceptance. The a* and b* values were changed from 5.54 ± 0.6 to 6.45 ± 0.7 and 39.45 ± 1.8 to 32.67 ± 2.3, respectively. These values indicate that extrusion process will change the color slightly to yellowish and reddish in comparison with USRB. Ultimately, the total color was improved by extrusion from 69.55 to 54.53, which is within proper color (light brown) for consumer acceptance (Tao et al., 1993).

### 3.3 Nutritional properties

The nutritional properties of USRB and SRB are provided in Table 2. The phytic acid content of USRB was 23.34 ± 0.23 mg/g, which was significantly lower than the other commercial rice bran, which can be related to the action of milling and separating of bran as well as the cultivar. Many researchers evaluated the phytic acid content of rice bran by different methods such as colorimetric and HPLC by Knuckles, Kuzmicky, and Betschart (1982) (54 and 78 mg/g), colorimetric method by Ravindran, Ravindran, and Sivalogan (1994) (36.5 mg/g), HPLC method by Kasim and Edwards (1998) (60 mg/g), and titration method by Garcia‐Estepa et al. (1999) (57.7 mg/g). In comparison with other cereals, the phytic acid of Tarom cultivar rice bran was similar to oat bran (21.5–24.0 mg/g). However, it was less than that of many varieties of wheat bran (25–47 mg/g) (Garcia‐Estepa et al., 1999). As can be found, the lowest phytic acid was found for Tarom cultivar rice bran (23 mg/g) that, to some extent, may be attributed to the place of cultivation and state of bran separation. The stabilized rice bran showed less phytic acid than the USRB and therefore more precise method, that is, colorimetric was used. The phytic acid content of SRB was 0.01 mg/g, which can be utilized for human consumption and iron anemia could be prevented by applying extrusion cooking. Indeed, extrusion cooking significantly reduced phytates in rice bran, and it can be concluded that the process can hopefully be applied to diminish the antinutritional matters in cereals such as rice bran. Besides, other analyses such as HLPC have shown that extrusion cooking or other thermal processing methods made the inositol hexaphosphate to hydrolyze as penta‐ and tetraphosphates, which cannot chelate the nutritional factors such as iron (Bullock, Duffin, & Nolan, 1993; Sandberg, Andersson, Carlsson, & Sandstrom, 1987). The results showed that niacin has the most quantity among B vitamins which were studied here. Moreover, the extrusion cooking did not have any statistical significant effect on B vitamins (*p* < 0.05), although vitamin E was significantly reduced by extrusion processing (*p* < 0.05).

**Table 2:** Nutritional proximate analysis of treated and untreated rice bran by extruder

Nutritional components	USRB	SRB
Phytate, mg/g	23.34 ± 0.23^a^	0.01 ± 0.01^b^
Vitamin E, mg/kg	1.60 ± 0.10^a^	1.1 ± 0.01^b^
Thiamine, mg/kg	0.10 ± 0.01^a^	0.09 ± 0.01^a^
Riboflavin, mg/kg	0.09 ± 0.01^a^	0.07 ± 0.01^a^
Niacin, mg/kg	0.34 ± 0.01^a^	0.31 ± 0.01^a^
Pantothenic acid, mg/kg	0.08 ± 0.01^a^	0.073 ± 0.01^a^
Folic acid, mg/kg	0.029 ± 0.01^a^	0.028 ± 0.01^a^

Statistical significant difference was presented in alphabetic order.

SRB, stabilized rice bran; USRB, unstabilized rice bran.

### 3.4 Functional properties

#### 3.4.1 Protein solubility

**Figure 1:**
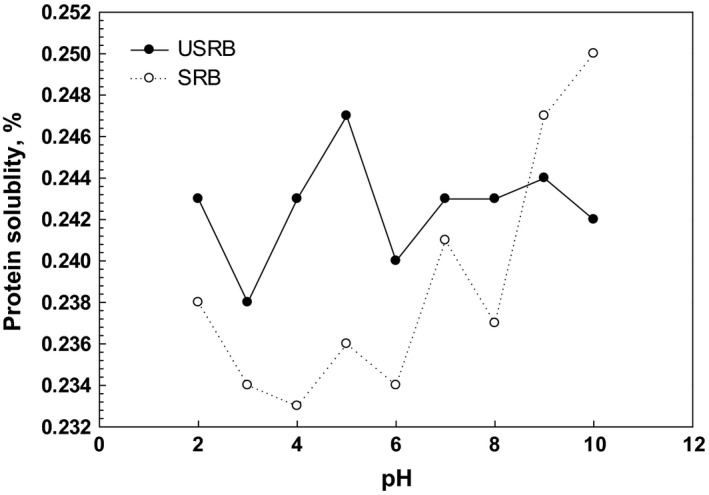
The protein solubility of unstabilized rice bran and stabilized rice bran at different pH levels (2.0–10.0)

Since the protein has a vital role in functional and structural properties of rice bran, its solubility was determined. The protein solubility is an important prerequisite in food protein functionality and is a good index for potential of protein applications. It has been reported that the protein solubility has a close relationship with emulsifying and foaming properties (Wang, Hettiarchchy, Qi, Burks, & Siebenmorgen, 1999). The protein solubility profiles of USRB and SRB at different pH (2.0–10.0) are shown in Figure 1. The lowest protein solubility for USRB and SRB was found at pH 4.0, which is near to the isoelectric point of protein (4.5). However, some fluctuation was seen, but both samples showed a gradual increase in solubility beyond the isoelectric pH and the protein solubility in aqueous solutions was dependent to pH value. Indeed, the acidic or alkaline conditions could increase the denaturation and hydrolysis of rice protein and therefore the solubility was enhanced (Esmaeili et al., 2016; Wang et al., 1999). At pH lower than 8.0, USRB showed higher solubility and after pH 8.0, the trend was changed and SRB showed more solubility. The statistical analysis of solubility showed that there is statistical significant difference between the solubility of USRB and the solubility of SRB at all pH levels (*p* < 0.05). On the other hand, SRB at alkaline conditions had higher protein solubility, which may be attributed to the main protein of rice, glutelin, that had high‐molecular weight and more solubility at higher pH (Ilankovan, Hettiarachchy, Christian, & Markus, 2007). Furthermore, it seems the protein solubility of rice bran can be affected by other components such as the dietary fiber, which it may be attached to the protein and improve its solubility. On the other hand, the soluble fraction of hemicelluloses, O‐acetyl galactoglucomannan, leads to good solubility in water and organic solvents (Lindblad, Albertsson, Ranucci, Laus, & Giani, 2005). Similar results were acquired for the solubility of unstabilized rice bran protein concentrate, which have been reported that the lowest solubility was found at pH 4.0 (Gnanasambandam & Hettiarachchy, 1995). Furthermore, hydrolysate powders of rice bran protein have shown higher nitrogen solubility (~ 98%) than that of SRB, which may be attributed to the smaller peptides (Kaewka, Therakulkait, & Cadwallader, 2009).

#### 3.4.2 Foaming capacity and stability

**Figure 2:**
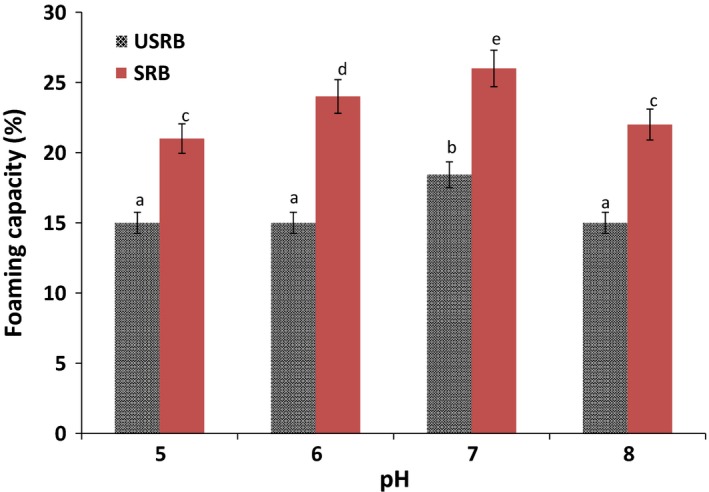
Effect of pH on foaming capacities of unstabilized rice bran and stabilized rice bran. Statistical significant difference was presented in alphabetic order.

In forming a stable air bubble, proteins have a significant role in which they should be dissolved in the aqueous phase and form a cohesive layer surrounding the gas/air droplets. Protein molecules should form continuous intermolecular polymer enveloping the air bubbles, since its intermolecular cohesiveness and elasticity are essential in producing the stable foams (Tang, Hettiarachchy, Horax, & Eswaranandam, 2003). Research has shown that pH has a significant effect on the volume and stability of foams (Esmaeili et al., 2016; Meuser, Busch, Fuhrmeister, & Rubach, 2001). In comparison with other proteins concerning foaming properties, egg albumin is the most dominant standard used in foaming ability (Symes, 1980). Foam capacities of raw and stabilized Tarom cultivar rice bran are given in Figure 2. It can be seen the foaming capacity of USRB was statistically unchanged at all pH levels except 7.0 (*p* < 0.05). Furthermore, by stabilization of rice bran, foaming capacity was improved more at all pH studied. However, the foaming capacity of USRB and SRB were less than rice bran proteins, which may be related to the loss of phytate, mineral, and cellular components in the extruded rice bran (Esmaeili et al., 2016). Similarly, foaming stability of USRB and SRB were negligible (Chandi & Sogi, 2007). Indeed, lack of ability of rice bran to foam stability is due to the fat and protein contents. If the fat is completely removed from rice bran, the foaming capacity and stability would be improved (James & Sloan, 1984). It was reported that good foaming capacity of rice bran protein is related to secondary and tertiary structure of rice bran proteins such as glutenin (Esmaeili et al., 2016).

#### 3.4.3 Water and oil absorption capacity

Water and oil absorption capacity play an important role in functional properties of rice bran for use in meat, bakery, and beverage industries. Results indicated that the water absorption capacity (WAC) of USRB (1.74 ± 0.09 g/g) was higher than that of rice bran protein (1.03 ± 0.006) (Esmaeili et al., 2016), which may be related to the high amount dietary fiber. In comparison with wheat bran (1 g/g), USRB showed more water absorption capacity (James & Sloan, 1984). Since the WAC is within the limit of 1.49–4.72 g/g, which is considered critical in viscous food such as soups and gravies (Aletor, Oshodi, & Ipinmoroti, 2002), it can be added to viscous products requiring high water content. The extrusion processing significantly improved the WAC of SRB to 2.56 ± 0.46 (*p* < 0.05). However, the WAC of SRB was less than previous work, which can be attributed to the different cultivars that they have used in their experiments (Chandi & Sogi, 2007; James & Sloan, 1984). In comparison with USRB, the water absorption capacity of SRB was improved as much as 20% indicating its ability to be utilized in foods requiring great amount of water such as sausages or baked goods. Therefore, SRB might be incorporated into these foods system for which a high water absorption capacity is desirable to assist with moisture, freshness, and softness characteristics.

High oil capacity is an important factor in formulation of food systems such as sausages or cake batters. Results showed that the oil absorption of USRB was 28.13% ± 0.03, which was less that of rice bran protein (87.33% ± 0.06) and whey protein (74.5%) (Esmaeili et al., 2016). Furthermore, the oil absorption capacity was less than bovine serum albumin (BSA), β‐lactoglobulin, casein, and ovalbumin (Voutsinas, Cheung, & Nakai, 1983). The high oil absorption capacity of USRB would be desirable in products such as meat extenders to help maintain juiciness and improve mouthfeel (Kinsella, 1982). Although USRB cannot be used in food systems such as donuts and pancakes that are cooked in frying oil, its high oil absorption would increase the oil usage and make oily goods. In contrary, the oil absorption capacity of SRB was obtained 17.80% ± 0.03, which was less than that of USRB. Therefore, it is an opportunity for SRB to exploit in the low‐calorie fried foods. Although rice bran protein concentrate/isolate have shown higher oil absorption capacity as compared with USRB, SRB, casein, various leaf protein concentrates, and soy protein isolates (Aletor et al., 2002; Esmaeili et al., 2016; Gandhi, Khare, & Jha, 2000).

## 4 Conclusion

Extrusion is a multistep thermal process, which is applied to a wide spectrum of food preparations. The effect of extrusion process on physicochemical, nutritional, and functional properties of Tarom cultivar rice bran was studied. The process reduced the protein in stabilized rice bran, which may be related to the denaturation of protein. The color of rice bran was improved by extrusion processing. Extrusion also had a reduced significant effect on the phytic acid as well as vitamin E of rice bran. However, it reduced niacin, riboflavin, pantothenic acid, and folic acid, but the dietary fiber was enhanced which has beneficial health effect on human health. In comparison with unstabilized rice bran, water‐holding capacity was enhanced, but oil absorption capacity was reduced. The foaming capacity/stability of extruded rice bran was better than that of USRB. However, these parameters were less than rice bran protein concentrate/isolate. Overall, the extrusion process improves some functional and nutritional properties of rice bran which are valuable in food applications and have potential ingredients in food in order to enhance consumer health. The results indicated that extruded rice bran can be successfully exploited for a variety of food formulations like weaning foods, dry mixes, baked foods, whipped toppings, salad dressing etc. due to their high foaming and emulsifying properties, as well as oil absorption and bulk density.

## Conflict of Interest

None declared.
